# Advances in the Effects of Dietary Macronutrients on the Gut Microbiota of Tilapia (*Oreochromis* spp.)

**DOI:** 10.3390/microorganisms12030543

**Published:** 2024-03-08

**Authors:** Weihao Ou, Zihe Guo, Ying Pan, Kai Huang, Yanqun Ma, Zhibiao Qin

**Affiliations:** Key Laboratory of Aquatic Healthy Breeding and Nutrition Regulation of Guangxi Universities, College of Animal Science and Technology, Guangxi University, Nanning 530004, China

**Keywords:** tilapia, proteins, lipids, carbohydrates, intestinal microbiota

## Abstract

The homeostasis of the intestinal microbiota of fish is beneficial to fish health, while food can affect the intestinal microbiota. Tilapia (*Oreochromis* spp.) has great economic value and is a good model to use in studying the digestion and absorption of nutrients. Furthermore, at present, due to a high demand and high price of high-quality feed raw materials, the nutritional composition of aquafeeds has been changing dynamically. There has yet to be a comprehensive review of research conducted on the influences of dietary macronutrients (proteins, lipids, and carbohydrates) on the tilapia intestinal microbiota. Therefore, this review focuses on the effects of dietary macronutrients on the gut microbiota of tilapia. Interestingly, we found that the best growth performance might not represent the best composition or functions of the gut microbiota. Overall, the unscientific addition of macronutrients to feed is harmful to the intestinal microbiota. Therefore, both growth performance and gut microbiota should be considered when evaluating certain macronutrients. It is our hope that this review will aid in regulating the intestinal microbiota of fish through nutritional means, thereby promoting tilapia farming.

## 1. Introduction

Tilapia (*Oreochromis* spp.) is an omnivorous fish with advantages such as delicious meat, rich nutrition, fast growth, and strong disease resistance [1,2,3]. Tilapia is a good model fish for nutrition and metabolism research because of its well-developed and well-characterized digestive and metabolic features as well as the availability of complete genomic information [4]. Moreover, tilapia is relatively easy to be farmed under various environmental conditions, is highly resistant to environmental stressors [5], and has been widely farmed worldwide. Tilapia has been one of the most important farmed finfish [6]. The tilapia industry can increase food security, nutritional value, and household incomes, thereby improving welfare in developing countries [2]. In recent years, the international market demand for tilapia has been increasing, being second only to trout in the global freshwater fish trade [1]. However, the productivity and profitability of tilapia aquaculture can be damaged by some diseases, such as viral, parasitic, and bacterial diseases [7].

Gut microbes are closely related to body health; for example, they play an important role in the host fish development, nutrient metabolism, immunity, and endocrine and nervous systems [8,9,10,11,12]. In a study on the tilapia gut microbiota, a meta-analysis indicated that the predominant bacteria at the phylum level include Proteobacteria, Fusobacteria, Actinobacteria, Firmicutes, and Bacteroidetes, while at the genus level, the predominant bacteria include *Cetobacterium*, *Lactobacillus*, *Legionella*, *Lactococcus*, *Rhodobacter*, *Pelomonas*, and *Streptococcus* [2]. It is well known that food composition can affect the gut microbiota. Reasonable food composition can optimize the intestinal microbiota to improve the digestion and absorption of nutrients, promote development, reduce the invasion of pathogenic microorganisms, and boost immunity. Currently, the price of some macronutrient sources is very high, especially fish meal and fish oil. To reduce costs and maximize production benefits, the contents and sources of dietary macronutrients in aquafeeds have been changing dynamically. However, the use of inappropriate contents and origins of dietary macronutrients destroys the gut microbiota, damaging fish health. Therefore, high-quality feeds are inseparable from a reasonable combination of dietary macronutrients, which is significant for the homeostasis of the gut microbiota, in order to improve disease resistance, health, and growth of tilapia.

Ringø et al. [13] have reviewed the effects of dietary components on the gut microbiota of aquatic animals. In addition, Haygood and Jha [14] have reviewed strategies to modulate the intestinal microbiota of tilapia in aquaculture, which included a brief introduction to the effects of alternative feed ingredients on the intestinal microbiota of tilapia. However, a more comprehensive review of the effects of dietary macronutrients on the intestinal microbiota of tilapia is needed in order to better regulate the intestinal microbiota of tilapia through nutritional methods. In light of the described features, this review examines the effects of dietary proteins, lipids, and carbohydrates on the intestinal microbiota of tilapia. This review is expected to provide research direction and assistance in future studies on the tilapia gut microbiota, thus contributing to the development and dissemination of scientific foundations in the tilapia production sector, including responsible breeding practices.

## 2. The Effects of Dietary Macronutrients on the Gut Microbiota of Tilapia

The dietary protein, lipid, and carbohydrate requirements of tilapia are 20–40% [15], 5–12% [16], and 30–40% [14], respectively. To the best of the authors’ knowledge, very little information is available on the effects of dietary macronutrients on the gut microbiota of tilapia. At present, most of the existing studies on the appropriate levels and sources of different dietary macronutrients of tilapia are mainly based on the growth performance of tilapia, ignoring the assessment of the impacts of dietary macronutrients on the intestinal microbiota, which can easily lead to the imbalance of intestinal microecology of tilapia and increase the risk of disease outbreak. In general, disordered functions, lower diversities, decreased abundance of beneficial microorganisms, and increased abundance of pathogenic or potentially pathogenic microorganisms of the intestinal microbiota are important characteristics of the disease state of fish. The following section focuses on the effects of dietary proteins (Table 1), lipids (Table 2), and carbohydrates (Table 3) on the gut microbiota of tilapia.

The exact mechanisms by which dietary macronutrients influence the gut microbiota are unclear. Three major mechanisms are likely to change the gut microbiota. Firstly, dietary macronutrients can provide nutrients for the gut microbiota, and different microbes have different nutritional metabolism abilities. In other words, a particular dietary macronutrient can promote the growth of some gut microbes that can take advantage of that dietary macronutrient. For example, the intestinal microbiota of farmed fish have a stronger function in carbohydrate metabolism compared with wild fish. This difference could be related to protein-rich diets for wild fish and carbohydrate-rich diets for farmed fish [17,18]. Lipid sources also regulate the fish gut microbiota, possibly because of the fact that different intestinal microbes use different lipid sources [19]. Secondly, some components of dietary macronutrients have direct bacteriostatic effects, such as fatty acids, which can inhibit bacteria through the lysis and solubilization of bacterial cell membranes and suppression of ATP production [20]. Some peptides also possess antimicrobial properties [21]. Thirdly, dietary macronutrients can affect the gut microbiota by altering host physiology, such as immunity. The nutritional immunity of the fish intestine has been extensively reviewed by Dawood [22].

**Table 1:** The effects of dietary proteins on the gut microbiota of tilapia.

Species	Initial Weight	Feeding Period	Diet Preparation	Methods for Analysis of the Intestinal Microbiota	Main Results	References
Nile tilapia (*Oreochromis niloticus*)	38.75 ± 0.61 g	56 days	Experimental diets contained 20% (low), 30% (moderate), and 40% (high) crude protein, respectively	Paired-end sequencing of the V3–V4 region of the microbial 16S rRNA gene amplicon was performed on the Illumina MiSeq platform	Dietary protein levels had no significant effect on microbial diversity but exerted an influence on microbial composition	Yang et al. [23]
GIFT tilapia (*Oreochromis niloticus*)	About 0.8 g	56 days	(1) NPD diet (35% protein) (2) LPD diet (25% protein)	Paired-end sequencing of the V3–V4 region of the microbial 16S rRNA gene amplicon was performed on the Illumina MiSeq platform	The LPD diet significantly lowered microbial diversity and increased the abundance of Bacteroides, while the NPD diet increased the abundance of *Lawsonia*, *Romboutsia*, and *Sphingomonas*	Zhu et al. [24]
Nile tilapia	6.16 ± 2.0 g	56 days	Sardine protein hydrolysates (SPHs) with different degrees of hydrolysis (low: 5.5%; intermediate: 27.9%; and high: 62.5%) were added to experimental diets to replace 5% of dietary protein, while the control diet was without SPH	Spread plate	Fish fed with the control diet had significantly highest total heterotrophic bacteria concentrations and highest Vibrionaceae concentrations, while no difference was observed among all groups for lactic acid bacteria concentrations	Ha et al. [25]
Nile tilapia	1.52 ± 0.6 g	56 days	Four diets with 0, 50, 100, and 190 g kg^−1^ of corn protein concentrate (CPC) as a replacement for fish meal	Spread plate	Compared with fish fed with diets containing 0 or 50 g kg^−1^ of CPC, fish fed with diets containing 100 or 190 g kg^−1^ of CPC had significantly lower total counts of aerobic heterotrophic bacteria and coliform	Khalifa et al. [26]
Nile tilapia	27.8 ± 0.5 g	5 months	Fish meal protein was replaced by rice protein concentrate (RPC) at rates of 0% (control), 25%, 50%, and 75%	Paired-end sequencing of the V3–V4 region of the microbial 16S rRNA gene amplicon was performed on the Illumina MiSeq platform	RPC-enriched diets changed the microbial community composition, decreased the microbial diversity, and increased the relative abundance of Bacteroidetes and Fusobacteria	Reda et al. [27]
Nile tilapia	8.2 ± 0.2 g	84 days	(1) Plant-protein-based diet (without fish meal) (2) Fish-protein-based diet (without soybean meal)	Spread plate	The counts of bacteria (*Bacillus* spp., *Vibrio* spp., *Pseudomonas* spp., and *Aeromonas* spp.) isolated from the intestinal content of Nile tilapia were not influenced by different dietary protein sources	Kishawy et al. [28]
Nile tilapia	0.66 g	56 days	(1) Diet A: high in fish meal (10.0%) and low in soybean meal (14.0%) (2) Diet B: low in fish meal (5.00%) and high in soybean meal (28.0%)	Paired-end sequencing of the V3–V4 region of the microbial 16S rRNA gene amplicon was performed on the Illumina HiSeq 2500 platform	Diet A and Diet B formed distinct clusters for both the allochthonous and autochthonous microbiota	Ran et al. [29]
Hybrid tilapia (*Oreochromis niloticus* ♀ × *Oreochromis aureus* ♂)	About 1.9 g	56 days	Based on the control (basal diet), completely hydrolyzed feather meal was used to replace isonitrogenous cottonseed meal or soybean meal with percentages of 6% and 12%	The V3 region of the microbial 16S rRNA gene was detected using PCR–DGGE (denaturing gradient gel electrophoresis)	The microbiota of fish fed with diets containing completely hydrolyzed feather meal clustered together, while the microbial cluster of fish fed with the control diet was far away from them	Zhang et al. [30]
Nile tilapia	12.09 ± 0.43 g	56 days	(1) SBM diet (470.0 g kg^−1^ soybean meal, 0 g kg^−1^ sorghum DDGS) (2) SBM/DDGS diet (329.0 g kg^−1^ soybean meal, 184.3 g kg^−1^ sorghum DDGS)	The V4 region of the microbial 16S rRNA gene amplicon was sequenced on the Illumina MiSeq platform	Partially replacing soybean meal with sorghum DDGS increased the microbial diversity and elevated the abundance of *Plesiomonas* and *Lactobacillus*	de Macedo et al. [31]
Nile tilapia	93.75 ± 5.57 g	30 days	A 5% fermented soybean meal (fermented by *Lactobacillus fermentum*, *Bacillus natto*, and *Saccharomyces cerevisiae*, respectively) was added to the control diet to partially replace soybean meal	Paired-end sequencing of the V3–V4 region of the microbial 16S rRNA gene amplicon was performed on the Illumina HiSeq 2500 platform	Dietary supplementation of fermented soybean meal increased the microbial diversity and the proportion of beneficial bacteria	Li et al. [32]
Nile tilapia	70.0 ± 4.0 g	56 days	The contents of *Moringa oleifera*, fermented *Moringa oleifera*, *Folium mori*, and fermented *Folium mori* meals in four different woody forage diets were 20%, 20%, 20%, and 20%, respectively, while the reference diet did not contain woody forages	Spread plate	A large number of aerobic culturable bacteria of fish showed a distinct antibacterial activity against some fish pathogens	Wu et al. [33]
Nile tilapia	70.0 ± 5.0 g	56 days	The contents of *Moringa oleifera*, fermented *Moringa oleifera*, *Folium mori*, and fermented *Folium mori* meals in four different woody forage diets were 20%, 20%, 10%, and 10%, respectively, while the reference diet did not contain woody forages	Paired-end sequencing of the V3–V4 region of the microbial 16S rRNA gene amplicon was performed on the Illumina HiSeq 2500 platform	Woody forages could effectively boost the diversity and difference in the microbial community and might affect the distribution of certain unique microbial communities	Zhang et al. [4]
Nile tilapia	12.0 ± 1.0 g	55 days	Diets containing *Moringa oleifera* Lam (MOL), *Folium mori* (FM), *Broussonetia papyrifera* (BP), or *Neolamarckia cadamba* (NC) were made with 70% of the reference diet and 30% of corresponding woody leaves	Paired-end sequencing of the V3–V4 region of the microbial 16S rRNA gene amplicon was performed on an Illumina platform	The diet containing 30% NC had negative effects on the microbial composition, but the diet containing 30% MOL had positive effects	Zeng et al. [34]
Nile tilapia	10 g	70 days	Experimental diets were supplemented with 4%, 8%, and 10% *Moringa oleifera* leaves, respectively, while the control diet did not contain *Moringa olifera* leaves	Pour plate and staining	*Moringa olifera* leaves decreased the abundance of pathogenic bacteria, for example, *Escherichia coli*, *Pseudomonas aeruginosa*, *Shigella*, and *Salmonella*	Parveen et al. [35]

**Table 2:** The effects of dietary lipids on the gut microbiota of tilapia.

Species	Initial Weight	Feeding Period	Diet Preparation	Methods for Analysis of the Intestinal Microbiota	Main Results	References
Nile tilapia (*Oreochromis niloticus*)	8.45 ± 0.15 g	65 days	Medium (70 g kg^−1^)- and high (120 g kg^−1^)-fat diets with or without oxytetracycline (2.00 g kg^−1^)	Paired-end sequencing of the V3–V4 region of the microbial 16S rRNA gene amplicon was performed on the Illumina HiSeq 2500 platform	The adverse effects of oxytetracycline on the microbiota of fish could be worsened by the high-fat diet	Limbu et al. [36]
Nile tilapia	5 g	56 days	(1) Control diet (5% soybean oil) (2) High-fat diet (11% soybean oil)	qPCR	The abundance of β-Proteobacteria, Actinobacteria, Enterobacteriaceae, and *Citrobacter* spp. in the control group was significantly lower than those in the high-fat group	Yi [37]
Nile tilapia	About 3~4 g	56 days	Using soybean oil as a lipid source, the basal diet (6.8% fat) and the high-fat diet (16.4% fat) with or without *Citrobacter freundii*	(1) Spread plate (2) qPCR (3) Sequencing of the V3–V4 region of the microbial 16S rRNA gene amplicon was performed on an Illumina platform	The high-fat diet favored the growth of Firmicutes and *Citrobacter* spp., but *Citrobacter* spp. were not abundant in the intestine	Zhang et al. [38]
Nile tilapia	4.5 ± 0.3 g	56 days	Four experimental diets with 15% lipid and different lipid sources, namely common mixed PL oil (60% palm oil + 40% linseed oil) (PLC), common mixed FL oil (60% fish oil + 40% linseed oil) (FLC), microencapsulated PL oil (PLM), and microencapsulated FL oil (FLM)	Sequencing of the V4–V5 region of the microbial 16S rRNA gene amplicon was performed on an Illumina platform	Microencapsulated oils as lipid sources in the high-fat diet improved the microbiota, and microencapsulated FL oil had less significant impact on the microbiota than microencapsulated PL oil	Ma et al. [39]
GIFT tilapia (*Oreochromis niloticus*)	13.73 ± 0.31 g	84 days	Four experimental diets containing fresh fish oil or highly oxidized fish oil either with or without ferulic acid (0 or 400 mg kg^−1^)	Paired-end sequencing of the V3–V4 region of the microbial 16S rRNA gene amplicon was performed on the Illumina MiSeq platform	Oxidized fish oil disturbed the microbiota	Yu et al. [40]
Hybrid tilapia (*Oreochromis niloticus* × *Oreochromis aureus*)	6.09 ± 0.07 g	63 days	Adding 0 or 400 mg kg^−1^ of tea polyphenols to three diets with 0, 15, and 30 g kg^−1^ of oxidized soybean oil (OSO)	Spread plate	Diets containing 15 or 30 g kg^−1^ of OSO significantly decreased total bacterial amount, and a diet containing 30 g kg^−1^ of OSO significantly reduced lactic acid bacteria amount and significantly raised *Escherichia coli* amount	Liang [41]

**Table 3:** The effects of dietary carbohydrates on the gut microbiota of tilapia.

Species	Initial Weight	Feeding Period	Diet Preparation	Methods for Analysis of the Intestinal Microbiota	Main Results	References
Nile tilapia (*Oreochromis niloticus*)	3.77 ± 0.01 g	56 days	(1) Low-starch diet (25% corn starch) (2) High-starch diet (45% corn starch)	Sequencing of the V4–V5 region of the microbial 16S rRNA gene amplicon was performed on an Illumina platform	The high-starch diet did not significantly change the microbiota	Liu [42]
Nile tilapia	1.19 ± 0.01 g	70 days	(1) Control diet (CON: 35% carbohydrate) (2) High-carbohydrate diet (HC: 45% carbohydrate) (3) Inulin-supplemented diet (HCI: 45% carbohydrate + 0.5% inulin)	Paired-end sequencing of the V3–V4 region of the microbial 16S rRNA gene amplicon was performed on the Illumina MiSeq platform	The high-carbohydrate diet decreased the abundance of Proteobacteria and Fusobacteria and increased the quantity of Actinobacteria	Wang et al. [43]
Nile tilapia	5.51 ± 0.16 g	56 days	(1) Control diet (CK: 30% carbohydrate) (2) High-carbohydrate diet (HC: 42% carbohydrate)	Paired-end sequencing of the V3–V4 region of the microbial 16S rRNA gene amplicon was performed on the Illumina MiSeq platform	The high-carbohydrate diet tended to raise the ratio of Proteobacteria, Firmicutes, and Chlamydiae but reduced the proportion of Actinobacteria and Fusobacteria	Wu et al. [44]
Nile tilapia	1.63 ± 0.05 g	70 days	(1) Control diet (30% maize starch) (2) High-carbohydrate diet (45% maize starch)	Paired-end sequencing of the V3–V4 region of the microbial 16S rRNA gene amplicon was performed on an Illumina platform	The high-carbohydrate diet significantly reduced the microbial diversity, significantly increased the abundance of Firmicutes, and significantly decreased some SCFA-producing bacteria	Xu et al. [45]
Nile tilapia	1.66 ± 0.05 g	70 days	(1) Normal control diet (NC: 30% corn starch) (2) High-carbohydrate diet (HC: 45% corn starch)	Paired-end sequencing of the V3–V4 region of the microbial 16S rRNA gene amplicon was performed on the Illumina MiSeq platform	The high-carbohydrate diet increased the abundance of Firmicutes but decreased the abundance of Bacteroidetes	Xu et al. [46]
Nile tilapia	2 ± 0.5 g	56 days	(1) Control diet (CON: 25% corn starch) (2) High-carbohydrate diet (HC: 45% corn starch)	Paired-end sequencing of the V3–V4 region of the microbial 16S rRNA gene amplicon was performed on the Illumina MiSeq platform	The high-carbohydrate diet did not change the microbial diversity but significantly increased the abundance of Actinobacteria	Li [47]
Nile tilapia	9.50 ± 0.08 g	35 days	Four experimental diets were prepared to contain medium carbohydrate (MC: 33.5% corn starch) and high carbohydrate (HC: 45.5% corn starch) with (MCO) or without (HCO) 0.2% oxytetracycline	Paired-end sequencing of the V3–V4 region of the microbial 16S rRNA gene amplicon was performed on the Illumina HiSeq 2500 platform	The high-carbohydrate diet could protect fish from side effects caused by oxytetracycline by inhibiting pathogenic bacteria and raising the proportion of beneficial bacteria	Limbu et al. [48]
Nile tilapia	100.60 ± 37.65 g	45 days	Five experimental diets contained different 30% carbohydrate sources (dextrin, ground corn, wheat bran, cassava bagasse, or broken rice)	(1) Spread plate (2) PCR–DGGE (the V3 region of the microbial 16S rRNA gene)	The count of amylolytic bacteria was the highest in the ground corn treatment and the lowest in the broken rice and wheat bran treatment	Pedrotti et al. [49]
Nile tilapia	9.81 ± 0.42 g	42 days	The ratios of amylose to amylopectin in five experimental diets were 0.10 (diet 1), 0.24 (diet 2), 0.47 (diet 3), 0.76 (diet 4), and 0.98 (diet 5), respectively	Spread plate	With the increase in the percentage of dietary amylose to amylopectin, the number of *Escherichia coli* and *Lactobacillus* spp. also increased	Wang et al. [50]

### 2.1. The Effects of Dietary Proteins on the Gut Microbiota of Tilapia

#### 2.1.1. Protein Levels

Proteins are the main cost of aquafeed production. To the best of our knowledge, only two studies have explored the effects of different dietary protein levels on the intestinal microbiota of tilapia. Compared with low (20%; LP group) dietary protein levels, moderate (30%; MP group) and high (40%; HP group) dietary protein levels improved the growth performance of Nile tilapia (*Oreochromis niloticus*) [23]. In addition, both dietary protein levels had no significant effect on the intestinal microbial diversity, but affected the intestinal microbial composition [23]. The HP group had the highest abundance of *Clostridium*, while the LP group had the highest abundance of *Enterovibrio* and *Grimontia* [23]. Genetically improved farmed tilapia (GIFT, *Oreochromis niloticus*) fed with a diet containing a low dietary protein level (25%) had significantly lower growth performance, significantly lower intestinal microbial diversity, and a higher abundance of *Bacteroides*, while fish fed with a diet containing a normal dietary protein level (35%) had plenty of *Lawsonia*, *Romboutsia*, and *Sphingomonas* [24].

#### 2.1.2. Substitutes for Fish Meal

Fish meal is the main dietary protein source due to its favorable nutritional qualities such as good digestibility and palatability, high protein content, great amino acid profile, and other essential nutrients [6,51]. However, the shortage, huge demand, and high price of fish meal have driven researchers to look for other abundant and cheaper protein sources to replace fish meal. Reviews concerning alternative dietary protein sources for tilapia include El-Sayed [52] and Montoya-Camacho et al. [53]. Compared with Nile tilapia fed with experimental diets containing sardine protein hydrolysates, Nile tilapia fed with the control diet without sardine protein hydrolysates had better growth performance, and higher total heterotrophic bacteria concentrations and Vibrionaceae concentrations in the gut, while no significant difference was observed in lactic acid bacteria concentrations in the gut among all groups [25]. The results of the evaluation of replacing fish meal with corn protein concentrate (CPC) in Nile tilapia showed that the growth performance was not significantly different in fish fed with diets containing 0, 50, and 100 g kg^−1^ of CPC, but were significantly decreased in fish fed with diets containing 190 g kg^−1^ of CPC [26]. Moreover, total counts of intestinal aerobic heterotrophic bacteria and coliform in fish fed with diets containing 100 or 190 g kg^−1^ of CPC were significantly lower than those in fish fed with diets containing 0 or 50 g kg^−1^ of CPC [26]. Diets containing rice protein concentrate as a replacement for fish meal protein changed the composition of the microbial community, decreased the microbial diversity, and increased the relative abundances of Bacteroidetes and Fusobacteria in the intestine of Nile tilapia [27].

Since soybean meal has high protein content, a relatively balanced amino acid profile, and good availability, it has been widely used as an alternative to fish meal [54,55]. However, information on the effects of replacing different levels of fish meal with soybean meal on the gut microbiota of tilapia is very limited. Two basal diets without fish meal (plant-protein-based diet) or soybean meal (fish-protein-based diet) had no significant effect on the growth performance and the bacteria counts (*Bacillus* spp., *Vibrio* spp., *Pseudomonas* spp., and *Aeromonas* spp.), which were isolated from the intestinal content of Nile tilapia [28]. However, the growth performance of Nile tilapia fed with Diet A (high in fish meal and low in soybean meal) was significantly worse than that of Nile tilapia fed with Diet B (low in fish meal and high in soybean meal), and both diets formed distinct clusters for both the intestinal allochthonous and autochthonous microbiota [29]. There are many reasons for these contradictory results, including not only differences in methods for analyzing the gut microbiota (spread plate for Kishawy et al. [28] and high-throughput sequencing for Ran et al. [29]) but also differences in fish genetics, rearing conditions, and feed formulations, etc. However, the detailed mechanisms need further research.

#### 2.1.3. Substitutes for Soybean Meal

Soybean meal is the most commonly used plant protein source in aquafeeds, and the price of soybean meal has become more expensive because of the high environmental cost of soybean production [56] and the food–feed competition caused by the increasing demand for soybean from humans and farmed animals [51]. Moreover, the presence of anti-nutritional factors is usually considered one of the main disadvantages of soybean meal [54]. Therefore, replacing soybean meal or improving the availability of soybean meal is an important problem. In hybrid tilapia (*Oreochromis niloticus* ♀ × *Oreochromis aureus* ♂), according to the principal component analysis, the gut microbiota of fish fed with diets containing completely hydrolyzed feather meal as a partial replacement for soybean meal or cottonseed meal clustered together, while the gut microbiota of fish fed with the control diet were far away from them; however, none of the diets had a significant effect on the growth performance [30]. In Nile tilapia, partially replacing soybean meal with sorghum DDGS (sorghum distillers’ dried grains with solubles) increased the diversity of the intestinal microbiota and elevated the abundance of intestinal beneficial bacteria *Plesiomonas* and *Lactobacillus*, but significantly decreased the growth performance [31].

It is well known that fermentation can improve the nutritional value and availability of soybean meal. Li et al. [32] reported that partially replacing soybean meal with fermented soybean meal (fermented using *Lactobacillus fermentum*, *Bacillus natto*, and *Saccharomyces cerevisiae*, respectively) significantly improved the growth performance and increased microbial diversity and the proportion of beneficial bacteria in the intestine of Nile tilapia. Intriguingly, Li et al. [32] also reported that dietary *Lactobacillus fermentum*- or *Saccharomyces cerevisiae*-fermented soybean meal would probably affect several microbial members who play a key role in the co-occurrence network and are more inclined to activate similar “big team” species and facilitate their cooperation to fulfill some functions in the intestine of Nile tilapia.

#### 2.1.4. Woody Forages

Several woody plants, low in cost and high in nutritional value, have attracted more and more attention as ideal new feed raw materials for aquatic animals [34]. Wu et al. [33] reported that a large number of aerobic culturable intestinal bacteria of Nile tilapia fed with diets containing different woody forages (*Moringa oleifera*, fermented *Moringa oleifera*, *Folium mori*, and fermented *Folium mori* meals) showed a distinct antibacterial activity against some fish pathogens. The study of Zhang et al. [4] demonstrated that woody forages (*Moringa oleifera*, fermented *Moringa oleifera*, *Folium mori*, and fermented *Folium mori* meals) could effectively boost the diversity and difference in the intestinal microbial community of Nile tilapia; moreover, they speculated that woody forages might affect the distribution of certain unique microbial communities (*Desulfovibrio*, *Thiobacillus*, *Methylobacillus*, *Marmoricola*, and *Syntrophobacter*) because two predicted functional pathways of the intestinal microbiota—membrane transport and carbon metabolism—in fish fed with the reference diet were significantly lower than those in fish fed with diets containing woody forages. In a previous study, feeds containing woody leaves of *Moringa oleifera* Lam (MOL), *Folium mori* (FM), *Broussonetia papyrifera* (BP), or *Neolamarckia cadamba* (NC) were fed to Nile tilapia, and the results showed that adding 30% NC to feed had negative effects on the gut microbiota and the growth performance, but adding 30% MOL to feed brought positive effects on the gut microbiota and the growth performance [34]. Parveen et al. [35] reported that dietary *Moringa oleifera* leaves significantly reduced the abundance of intestinal pathogenic bacteria *Escherichia coli*, *Pseudomonas aeruginosa*, *Shigella*, and *Salmonella* and significantly increased the growth performance of Nile tilapia.

### 2.2. The Effects of Dietary Lipids on the Gut Microbiota of Tilapia

Lipid is known as one of the most important energy sources. Because of its low price, compared to protein, researchers and commercial feed manufacturers have tried to increase lipid content and reduce protein in feeds. However, carefully preparing species-specific diets is recommended, as using high-lipid levels or inappropriate lipid sources is known to impair the gut microbiota and cause nutritional pathologies in fish [57,58]. In addition, in aquafeed production and storage processes, lipids in aquafeeds are easily oxidized, especially in feeds containing high doses of lipids or unsaturated fatty acids. Oxidized lipids can also endanger the intestinal microbiota of fish [59].

#### 2.2.1. Lipid Levels

Limbu et al. [36] reported that the high-fat diet could aggravate the adverse effects of oxytetracycline on the intestinal microbiota and the growth performance of Nile tilapia. Through the prediction of the gut microbiota functional pathways, compared with the medium-fat diet, they found that the high-fat diet significantly diminished the metabolism pathway, genetic information processing pathway, and organismal systems pathway of the gut microbiota of Nile tilapia [36]. Intriguingly, the correlation analysis revealed that some gut bacterial species executed dissimilar functions at the two fat contents with oxytetracycline [36]. For example, *Plesiomanas*, which played detrimental roles in MF (medium-fat) vs. MFO (medium-fat with oxytetracycline) diets, such as decreasing growth performance and raising oxidative stress, endoplasmic reticulum stress, and apoptosis, conducted beneficial functions in HF (high-fat) vs. HFO (high-fat with oxytetracycline) diets. Conversely, *Macellibacteroidetes*, which conducted advantageous functions in MF vs. MFO diets, executed adverse functions in HF vs. HFO diets, but *Cytophagales*, which had no correlation with any function in MF vs. MFO diets, executed beneficial functions in HF vs. HFO diets.

Yi [37] stated that Nile tilapia fed with the high-fat diet had higher levels of β-Proteobacteria, Actinobacteria, Enterobacteriaceae, and *Citrobacter* spp. in the intestine. More specifically, also in the intestine of Nile tilapia, Zhang et al. [38] claimed that the high-fat diet reduced the abundances of *Bifidobacterium*, *Nocardia*, *Microbacteriaceae*, *Enterococcus*, *Lactobacillus*, *Lactococcus*, *Leuconostoc*, *Streptococcus*, and *Ralstonia*, as well as raised the abundances of Firmicutes, *Paraclostridium*, *Gemmobacter*, *Rhodobacter*, *Clostridium*, and *Aquicella*. In addition, Zhang et al. [38] also found that the high-fat diet favored the growth of *Citrobacter* sp. S1, which could help the host tilapia harvest more energy from the high-fat diet, but the proportion of *Citrobacter* sp. S1 was low in the intestine, indicating that rare microbes can play a crucial role in the physiological activities of the host. Interestingly, in the studies of Yi [37] and Zhang et al. [38], it was found that the high-fat diet could promote tilapia growth to a certain extent, but the high-fat diet often had harmful effects on the gut microbiota. Therefore, both the growth performance and the gut microbiota should be considered when evaluating certain macronutrients.

#### 2.2.2. Lipid Sources

Some previous studies have explored ways to reduce the intestinal microbial disorders caused by high-fat diets. Ma et al. [39] reported that Nile tilapia fed with the high-fat diet containing microencapsulated oils as lipid sources had more abundance of beneficial bacteria (*Bacillus* and *Paenibacillus*) and less quantity of harmful bacteria (*Pseudoalteromonas* and *Roseovarius*) in the gut, indicating that microencapsulated oils can improve the intestinal microbiota of fish; however, microencapsulated FL oil (60% fish oil + 40% linseed oil) had less significant impact on the intestinal microbiota than microencapsulated PL oil (60% palm oil + 40% linseed oil), suggesting that the effects of microencapsulated oil were related to lipid sources or fatty acid composition. In addition, microencapsulated oils did not significantly influence the growth performance of Nile tilapia [39]. It is hard to explain why microencapsulated oil positively affects the gut microbiota. Ma et al. [39] speculated that the increased time for dietary oil to pass through the digestive tract and the corresponding increase in dietary oil digestibility caused by feeding microencapsulated oil might favor the beneficial alterations of the intestinal microbiota. In addition, oil oxidation could be effectively retarded by microencapsulation [60,61], which might also benefit the intestinal microbiota. However, further studies are required to determine the details.

Yu et al. [40] pointed out that oxidized fish oil could disturb the intestinal microbiota and reduce the growth performance of GIFT tilapia. Oxidized fish oil significantly elevated the abundance of phylum Proteobacteria, and the increased abundance of Proteobacteria was tightly associated with gut microbiota dysbiosis and diseases [40]. Moreover, the research of Liang [41] showed that hybrid tilapia (*Oreochromis niloticus* × *Oreochromis aureus*) fed with diets containing 15 or 30 g kg^−1^ of oxidized soybean oil had a significantly lower intestinal total bacterial amount, and hybrid tilapia fed with a diet containing 30 g kg^−1^ of oxidized soybean oil had significantly poor growth performance, a significantly lower lactic acid bacteria amount, and a significantly higher *Escherichia coli* amount.

### 2.3. The Effects of Dietary Carbohydrates on the Gut Microbiota of Tilapia

Carbohydrates, the most abundant and cheapest non-protein energy sources, are becoming increasingly important and widely used in aquafeeds to reduce the use of proteins [62,63]. However, high doses of carbohydrates in feeds can pose health hazards to teleost [64,65,66,67,68] because fish use carbohydrates poorly. Further details on dietary carbohydrate utilization by tilapia can be found in the review by Maas et al. [62].

#### 2.3.1. Carbohydrate Levels

There are some contradictions in many studies about the effects of high dietary carbohydrates on the intestinal microbiota of tilapia. The studies of Liu [42], Wang et al. [43], Wu et al. [44], Xu et al. [45], Xu et al. [46], and Li [47] mentioned below have shown that the high-carbohydrate diet increased the body weight of tilapia, but the composition or functions of the intestinal microbiota might not be optimal in the high-carbohydrate group. The study of Wang et al. [43] showed that the high-carbohydrate diet decreased the abundance of Proteobacteria and Fusobacteria, but increased the quantity of Actinobacteria in the intestine of Nile tilapia. The research of Wu et al. [44] showed that the high-carbohydrate diet tended to raise the ratio of Proteobacteria, Firmicutes, and Chlamydiae, but reduced the proportion of Actinobacteria and Fusobacteria in the intestine of Nile tilapia. Xu et al. [46] pointed out that the high-carbohydrate diet increased the abundance of Firmicutes but decreased the abundance of Bacteroidetes in the intestine of Nile tilapia. Xu et al. [45] also reported that the high-carbohydrate diet significantly reduced microbial diversity, significantly increased the abundance of Firmicutes, and significantly decreased some SCFA (short-chain fatty acid)-producing bacteria in the intestine of Nile tilapia. However, Liu [42] reported that the high-starch diet did not significantly change the intestinal microbiota of Nile tilapia. Li [47] also found that the high-carbohydrate diet did not change the microbial diversity, but significantly increased the abundance of Actinobacteria in the intestine of Nile tilapia. Therefore, as mentioned in Section 2.2.1. Lipid Levels, both the growth performance and the gut microbiota should be considered when evaluating certain macronutrients.

Interestingly, Limbu et al. [48] reported that the high-carbohydrate diet could protect Nile tilapia from side effects caused by oxytetracycline by inhibiting pathogenic bacteria and raising the proportion of beneficial bacteria in the intestine. In addition, dietary carbohydrate levels did not influence oxytetracycline-caused side effects on the growth performance of Nile tilapia [48]. These results contradict the study of Limbu et al. [36], which pointed out that the adverse effects of oxytetracycline on the intestinal microbiota and the growth performance of Nile tilapia could be exacerbated by the high-fat diet. Albeit the exact mechanisms exploited by carbohydrates to bind antibiotics are still unknown, Limbu et al. [48] inferred that the benefits of high doses of carbohydrates may be due to the ability of carbohydrates to bind antibiotics, which could result in lower bioavailability of oxytetracycline absorbed by the intestine and slower speed of absorption and transport of oxytetracycline.

#### 2.3.2. Carbohydrate Sources

A previous study reported that different dietary carbohydrate sources (dextrin, ground corn, wheat bran, cassava bagasse, or broken rice) could change the intestinal bacterial composition of Nile tilapia [49]. More specifically, the count of intestinal amylolytic bacteria was the highest in the ground corn treatment and the lowest in the broken rice and wheat bran treatment [49]. Moreover, Wang et al. [50] fed Nile tilapia with diets containing different ratios of amylose to amylopectin (0.10, 0.24, 0.47, 0.76, and 0.98, respectively), and they found that with the increase in the percentage of dietary amylose to amylopectin, the number of *Escherichia coli* and *Lactobacillus* spp. also increased in the intestine of Nile tilapia. Moreover, Wang et al. [50] also reported that the growth performance of Nile tilapia fed with diets containing different ratios of amylose to amylopectin was not consistent with the fermentation of their intestinal bacteria, which once again indicated that the best growth performance might not represent the best composition or functions of the gut microbiota. Therefore, when evaluating certain macronutrients, we should not only pay attention to the growth performance but also consider the gut microbiota.

## 3. Future Perspectives

### 3.1. Gnotobiotic Tilapia

Various influencing factors can perplex researchers of host–microbe interactions that induce challenges in related experiments such as repeatability and reproducibility deficiency. Gnotobiotic models, defined as animals reared under axenic conditions or with described microbial species [69], provide a critical tactic to reduce these troubles to a certain extent and have been widely used for determining the relationship between nutrients, the intestinal microbiota, and the host. However, the construction and development of gnotobiotic tilapia models are still very backward, which somewhat hinders the study of the nutritional physiology of tilapia. To the best of our knowledge, only three studies have used gnotobiotic tilapia models [5,70,71]. The existing gnotobiotic tilapia models have the following disadvantages. Hence, in the future, we should perfect gnotobiotic tilapia models and use them to study the interactions between nutrients, the gut microbiota, and the host in order to help regulate the intestinal microbiota of tilapia through nutritional approaches.

(1) The method of preserving gnotobiotic conditions is minimal and has great defects. Moreover, the three current studies used tilapia larvae because preserving gnotobiotic conditions for a long time is challenging. Thus, ensuring the preservation of the multigenerational lines of gnotobiotic tilapia models is critical.
(2) The means of detecting the gnotobiotic state need to be improved. For example, for the commonly used high-throughput sequencing analysis of 16S rRNA genes, a negative control and positive control should be set up in the process of sample preparation, PCR, library construction, and sequencing to exclude the possibility of contamination. In addition, a combination of some proper techniques (e.g., cultivation, staining, and high-throughput sequencing) rather than a single method should be used to monitor contaminations periodically.
(3) There still needs to be a relevant report on the specific nutritional requirements of gnotobiotic tilapia. Therefore, providing gnotobiotic tilapia with adequate nutrition is an intractable challenge.

### 3.2. Analysis Methods

In the future, we should flexibly apply various methods to study the effects of dietary macronutrients on the intestinal microbiota of tilapia and avoid using a single method as much as possible. Both culture-independent and culture-dependent methods are critical because different ways have different advantages and limitations, and the combination of various methods can obtain a more comprehensive profile of the gut microbiota. For example, high-throughput sequencing has a large amount of data, but detecting rare microorganisms is limited. On the other hand, qPCR has a relatively small amount of data, but this method is more sensitive and can detect some rare microbes; in particular, qPCR for microbes with specific functions can provide a deeper understanding of the roles of the intestinal microbiota. The recently reported Microbe-seq, a high-throughput approach that acquires the genomes of individual microbes from complex microbial communities, is expected to further promote microbial research with single-microbe resolution [72]. In addition, the traditional spread plate method can obtain particular strains, although less than 0.1% of the intestinal microbes of some fish could be cultured [73]. In recent years, the rapid development and continuous optimization of culturomics have promoted the acquisition and identification of many strains [74,75].

### 3.3. Microbial Types and Functions

Future research on the effects of dietary macronutrients on the tilapia gut microbiota should not only focus on bacteria, but also on other microorganisms, such as viruses and fungi, which will greatly contribute to related studies. Moreover, the existing studies mainly concentrated on the composition and diversity of the intestinal microbiota. Therefore, to better understand the interactions among macronutrients, the intestinal microbiota, and the host, it is also necessary to strengthen the study of microbial functions in the intestine.

## 4. Conclusions

**Figure 1:**
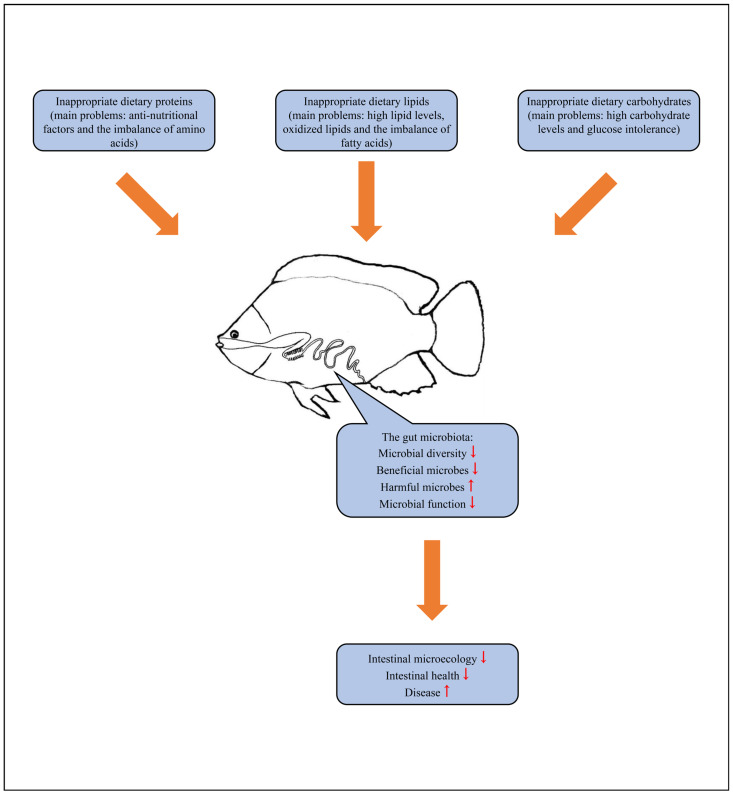
The main problems caused by improper applications of dietary macronutrients in tilapia and the resulting adverse effects on the gut microbiota of tilapia.

In conclusion, sources of dietary macronutrients need to be increased. Overall, the problems caused by improper applications of dietary macronutrients in tilapia mainly include the following: (1) anti-nutritional factors and the imbalance of amino acids in inappropriate dietary proteins; (2) high lipid levels, oxidized lipids, and the imbalance of fatty acids in inappropriate dietary lipids; and (3) high carbohydrate levels and glucose intolerance in inappropriate dietary carbohydrates. Negative effects on the gut microbiota of tilapia caused by these inappropriate applications of dietary macronutrients mainly include a reduction in microbial diversity, a decrease in the abundance of beneficial microbes, an increase in the proportion of harmful microbes, and dysfunction of the microbiota, resulting in the imbalance of the intestinal microecology, the destruction of intestinal health, and frequent diseases (Figure 1).

At present, the replacement of fish meal is still one of the focal points and difficulties in aquaculture, but the optimal substitution levels obtained in existing studies are more based on growth performance, and little attention is paid to the gut microbiota. Moreover, the best growth performance might not represent the best composition or functions of the gut microbiota. However, on the whole, the unscientific addition of macronutrients to feed is harmful to the intestinal microbiota. Therefore, both growth performance and gut microbiota should be considered when evaluating certain macronutrients. This approach will help us better regulate the gut microbiota to improve the utilization and tolerance of fish to different dietary macronutrients, thus improving economic benefits and promoting the healthy and sustainable development of aquaculture.

## Data Availability

No data were used for the research described in the article.
